# Self-assembled bovine serum albumin nanoparticles as pesticide delivery vectors for controlling trunk-boring pests

**DOI:** 10.1186/s12951-020-00725-z

**Published:** 2020-11-10

**Authors:** Chenyu Su, Shanshan Liu, Shenghan Cao, Shuyan Yin, Chenggang Zhou, Shangkun Gao, Chunyan Jia, Yingchao Ji, Yanxue Liu

**Affiliations:** 1 College of Plant Protection, Shandong Agricultural University, Tai’an, 271018 Shandong People’s Republic of China; 2 Shandong Research Center for Forestry Harmful Biological Control Engineering and Technology, Shandong Agricultural University, Tai’an, 271018 Shandong People’s Republic of China; 3 College of Animal and Veterinary Medicine, Shandong Agricultural University, Tai’an, 271018 Shandong People’s Republic of China; 4 Taishan Scenery and Scenic Spot Area Management Committee, Tai’an, 271000 Shandong People’s Republic of China

**Keywords:** Trunk-boring pests, Nano-pesticide, Self-assembly, Bovine serum albumin, Thiacloprid

## Abstract

**Background:**

Trunk-boring pests (TBPs) are an important type of forest pest, TBPs not only feed on the branches and trunks of trees, but also spread quarantine diseases in forests. However, because the larvae of TBPs live inside the trunk and are well concealed, prevention and control are difficult. The lack of effective control methods leads to the death of many trees in forests. In this study, a novel nanopesticide featuring high bioactivity and slow-release properties was developed to control TBPs. Thiacloprid (THI), which is commonly used to control *Coleoptera* species, was used as a model pesticide.

**Results:**

The oleophobic properties of bovine serum albumin (BSA) were exploited to encapsulate the hydrophobic pesticide THI by self-assembly, and the size of the obtained nanoparticles, THI@BSA·NPs, was approximately 23 nm. The loading efficiency reached 70.4%, and THI@BSA·NPs could be released continuously for over 15 days, with the cumulative release reaching 93.5%. The fluorescein isothiocyanate (FITC)-labeled nanoparticles were evenly distributed in the digestive tract and body surface of a typical TBPs, *M. alternatus*, and the stomach and contact toxicities increased by 33.7% and 25.9%, respectively, compared with those of free THI. Furthermore, the results showed that the transport efficiency of THI@BSA·NPs was highest at a concentration of 50 μg/mL, and the THI@BSA·NPs content in the trunk, from to lower to higher layers, was 8.8, 8.2, 7.6, and 5.8 μg/g. At the same time, THI@BSA·NPs also exhibited high transport efficiency in dead trees.

**Conclusion:**

The transport efficiency and toxicity of the active ingredients are the key factors for the control of TBPs. This work provided idea for the application of biological delivery system encapsulated hydrophobic pesticides. The novel self-assembled THI@BSA·NPs have promising potential for sustainable control of TBPs.
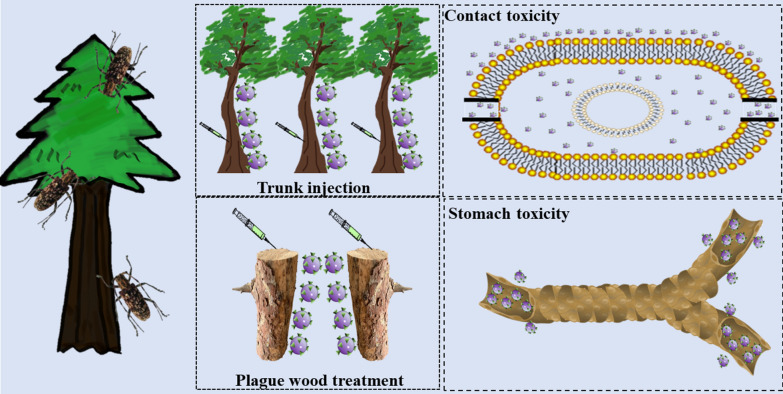

## Introduction

There are many species of trunk-boring pests (TBPs), including Cerambycidae, Buprestidae, and Scolytidae species [1–4]. The larvae of TBPs generally live inside the branches and trunkes of trees, affecting nutrient and water transport and even causing the trees to be riddled with holes, leading eventually to tree death [5]. Importantly, some TBPs can also spread plant diseases; for example, *Monochamus alternatus* is a quarantine pest and an important insect vector of the devastating forestry disease, pine wilt disease, known as a cancer or plague of pine trees [6, 7]. Pine wilt disease harms major species of *Pinus,* such as Scots pine (*P. sylvestris*), black pine (*P. thunbergii*), and Japanese red pine (*P. densiflora*), in East Asia, Europe, and North America [8]. Since pine wilt disease was introduced into China in 1982, billions of pine trees have been infected and have died, causing direct economic losses of hundreds of billions yuan [9, 10]. However, because TBPs are well concealed and live inside the branches and trunks, the use of traditional control methods is challenging; moreover, treatment of plague wood in high-altitude areas also leads to problems in forest protection. Currently, research is being conducted on the injection of pesticides into trees to control TBPs [11, 12], but the effect is not ideal; therefore, there is an urgent need to develop an efficient solution.

Nanotechnology is an emerging technology, and due to its good biocompatibility [13, 14], moderate delivery efficiency [15, 16], and outstanding sustained release performance [17, 18] and stability, it has been widely applied in energy conversion and storage [19, 20], microelectronics [21], precision biology [22, 23], environmental pollution remediation, the catalyst industry and membrane engineering. In particular, multifunctional nanotechnologies, such as nanosensing systems [24], gene targeting, nanooptics [25], precise and intelligent controlled release, and biological detection, have brought vitality to many fields. In particular, it should be pointed out that nanotechnologies are being developed for application in agriculture and forestry, and related reports are available. Li [26] used lignin, a biomaterial, to encapsulate avermectin to prevent avermectin from undergoing photodegradation. The encapsulation efficiency value reached 63%, and the half-life of avermectin under UV irradiation was prolonged by 7.5 times compared to that of uncoated avermectin. Liang reported that when prochloraz was encapsulated within the pores of mesoporous silica nanoparticles, the loading efficiency reached 25.4%, and the toxicity of the nanoparticles toward zebrafish decreased more than sixfold compared with that of prochloraz [27]. To solve the problem of pest resistance to Bt toxins, Zheng developed a nanoscale star polymer as a carrier for the pesticide Bt toxin Cry1Ab. This nanocarrier could effectively deliver Bt toxin to kill Bt-resistant pests. The toxicity toward Bt-resistant pests was significantly improved. The survival ratios of the 0.1 mg, 1 mg and 10 μg groups were reduced to 66.7%. 53.3% and 25.0% [28]. However, there are very few reports of the use of nanotechnology in forestry pest control, especially given the urgent need to develop new pesticide loading technologies to effectively control the severe economic losses caused by TBPs.

**Fig. 1:**
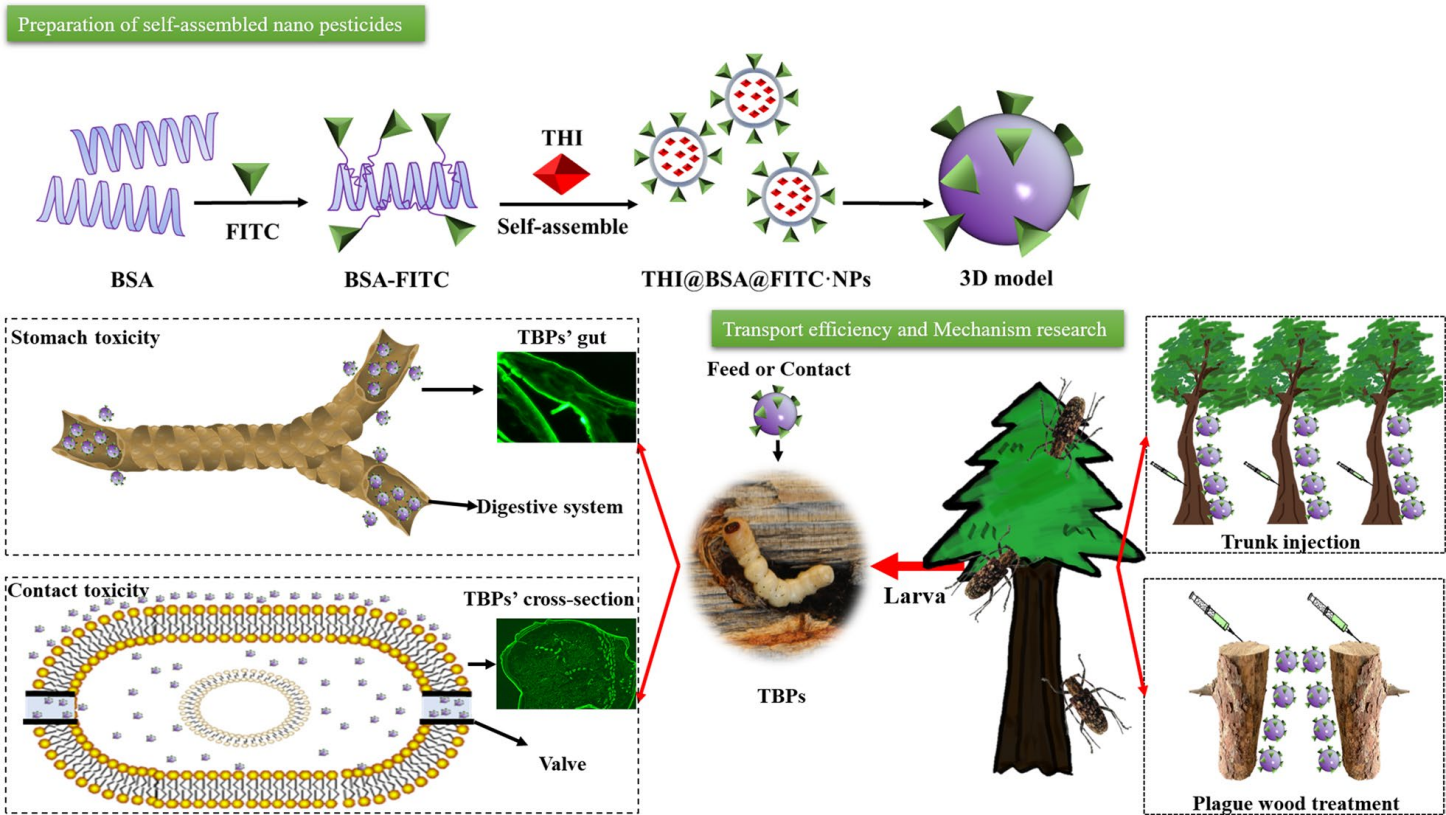
Schematic illustration of the THI@BSA·NPs, it can encapsulate the hydrophobic drug THI and is labeled with fluorescein isothiocyanate for pest control, for the transport of nanoparticles in the trunk of pine tree, and the contact and stomach toxicity mechanism. The fluorescent image of THI@BSA@FITC·NPs on larval surface, and the fluorescent distribution of THI@BSA@FITC·NPs on larval digestive system

In this study, using a polymer biomaterial with excellent biocompatibility, bovine serum albumin (BSA), was used as a pesticide carrier [29, 30]. A high-loading-efficiency BSA nanopesticide with high bioactivity was developed through self-assembly to control TBPs (Fig. 1). Thiacloprid (THI) ((3-((6-chloro-3-pyridyl) methyl)-1,3-thiazoline-2-ylidene) cyanamide) is a kind of neonicotinoid insecticide [31] that is mainly used in the control of TBPs [32–34]; therefore, THI was selected as the model pesticide in this study. First, a saturated BSA solution was added dropwise into THI acetonitrile solution and stirred at high speed to prepare THI bovine serum albumin nanoparticles (THI@BSA·NPs) by self-assembly. The size distribution and structure of THI@BSA·NPs were examined by dynamic light scattering (DLS) and scanning electron microscopy (SEM). The loading efficiency of THI@BSA·NPs was determined with different THI concentrations. The release behavior was also studied. Furthermore, the typical TBP and important vector insect of pine wilt disease, Monochamus alternatus, was chosen as the test insect, and the contact toxicity and stomach toxicity of THI@BSA·NPs to the 3rd instar larvae were compared. Then, by utilizing fluorescent staining technology, FITC was used to label BSA to synthesize fluorescent THI@BSA@FITC·NPs to explore the mechanisms of stomach toxicity and contact toxicity. Finally, by using pine trees as research objects, a simple comparison of the transport efficiency of THI@BSA·NPs in live trees was performed. Moreover, to address plague wood in high-altitude areas, THI@BSA·NPs application was considered to reduce the cost of disposal and the risk of infection. The transport efficiency of THI@ BSA·NPs in dead tree trunks was also studied.

## Materials and methods

### Materials

Biotech-grade (purity = 99%) of BSA was provided by Coolaber Science & Technology Co., Ltd (Beijing, China). Analytical-grade (purity = 97%) of thiacloprid (THI) powder was purchased from Aladdin Indusrial Corporation (Shanghai, China). Acetonitrile (analytical grade) was obtained from Kermel Chemical Reagent Co., Ltd. (Tianjin, China). The THI microcapsule suspension (THI@M) (purity = 2%) was obtained from Hailir Pesticides and Chemicals Group Co.; Ltd. (Qingdao, China). PBS (1 × , pH 7.2–7.4, 0.01 M), the conjugation-grade FITC (purity > 95%) and BS-grade Coomassie brilliant blue G-250 (Em (595 nm, H_2_O) > 36,300) were provided by Coolaber Science & Technology Co., Ltd (Beijing, China). Sodium chloride (purity > 99.5%) and anhydrous magnesium sulfate (analytical grade) were purchased from Shanghai Macklin Biochemical Co., Ltd (Shanghai, China). N-propyl ethylenediamine (PSA) was provided by Bonna-Agela Technologies Co., Ltd (Tianjin, China).

### Preparation of THI@BSA·NPs

The THI@BSA·NPs were synthesized by using a previously reported method with minor modification [35]. First, BSA (0.02 g) was dissolved in 2 mL of purified water at 50 °C (10 mg/mL). THI (0.04 g) was dissolved in 10 mL of acetonitrile in a conical flask (4 mg/mL). Then, BSA solution was added dropwise into AVM acetonitrile solution under high-speed stirring; the mixture was stirred at 900 rpm for 20 min and placed at − 20 °C. After ten minutes of ultrasonic dispersion, the organic solvent was removed by rotary evaporation. Finally, the nanoparticle powder was obtained by freeze-drying for 48 h. PVP (2%) was added to realize redispersion when the nanoparticle powder was used. To obtain a high liquid ratio, 2 mL of saturated BSA solution was added dropwise to 40 mL of THI solution. Under high-speed stirring, BSA was insoluble in the organic solvent; BSA will underwent changes in structure, and the chain was bent and folded to reach a steady state. Finally, the BSA formed a spherical structure and encapsulated the THI. The morphologies of THI@BSA·NPs were examined by scanning electron microscopy (SEM). The size distribution of THI@BSA·NPs was measured by a laser particle size analyzer.

### THI@BSA·NPs labeled with FITC

Sodium chloride (8.775 g) was dissolved in 1 L of purified water (1 L, 0.15 M). For sodium carbonate-sodium bicarbonate buffer (100 mL, 0.15 M, pH 9.0), Na_2_CO_3_ solution (10 mL, 1.59 wt%) and NaHCO_3_ solution (90 mL, 1.26 wt%) were mixed, and the pH was adjusted to 9.0. The sodium chloride solution and buffer solution above were mixed at a ratio of 9:1 to dissolve BSA (5 mg/mL). FITC was added to the BSA solution and the mass ratio of BSA to FITC was 50:1. The resulting solution was cultured with shaking overnight at 4 °C in the dark. Finally, the solution was filtered in a dialysis bag and centrifuged at 5000×*g* for 30 min, which was repeated three times until no FITC was filtered out [36]. Synthesis of THI@BSA@FITC·NPs with FITC-BSA instead of aqueous phase.

### Loading efficiency of THI@BSA·NPs

Different amounts of THI were dissolved in 15 mL of acetonitrile as the oil phase. BSA solution (2 mL, 10 mg/mL) was used as the aqueous phase, and added dropwise into the oil phase under magnetic stirring at 800 rpm. The solvent in the system was evaporated to dryness, and then, 10 mL of acetonitrile was added to dissolve the unencapsulated THI and centrifuged at 8000 rpm for 5 min. The free THI content was analyzed by measuring the absorbance at 241 nm by using a UV spectrophotometer. The THI loading efficiency of THI@BSA·NPs was calculated according to the following equation [37]:

$${\text{THI drug loading efficiency }}\left( {\text{\% }} \right){ = }\left( {\frac{{\text{Weight of initial THI - Weight of free THI}}}{{{\text{Weight of THI@BSA.NPs}}}}} \right){{ \times 100}}$$

### Controlled release of THI@BSA·NPs

The method was modified based on previous studies [38]. THI@BSA·NPs were dispersed in 50 mL of PBS (pH 5.0, 7.0, 9.0). Then, the THI@BSA·NPs PBS buffer solutions were evenly divided into 10 portions and aliquoted into 10 mL centrifuge tubes. The centrifuge tubes were placed in a shaker with an oscillation rate of 150 rpm at 25 °C. To compare the release of THI-BSA at different temperatures, THI@BSA·NPs were dispersed in PBS (pH 7.0) and placed in a shaker at 15 and 35 °C. Then, samples were taken at different time points, and the solvent in each sample was evaporated to dryness. Acetonitrile (5 mL) was added to dissolve the released THI, and the THI content was analyzed by measuring the absorbance at 241 nm by the using a UV spectrophotometer. The release of THI@M was measured by the same method.

### Toxicity bioassay

The contact toxicity and stomach toxicity of THI@BSA·NPs toward MA were determined by the drug membrane contact method and feed mixed drug method, respectively, with modifications [39, 40]. First, THI@BSA·NPs were dispersed in water and diluted to five gradient concentrations, and then, well-developed 3rd instar larvae of MA were picked. The drug membrane contact method was as follows: filter paper was wetted with each diluted solution, and 3rd instar larvae of MA were placed on the filter paper and allowed to crawl so that they came in full contact with the agent on the surface. After the treatment, the larvae were placed into 6-cm^3^ breeding boxes with artificial feed. Ten larvae were treated in each group, and treatment with each concentration was repeated three times, with the water treatment as the control group. The feed mixed drug method was as follows: before the experiment, 3 g of artificial feed was weighed and placed into breeding boxes, and diluted THI@BSA·NPs solution (1 mL) was added. Well-developed larvae were added to each breeding box. Ten larvae were treated in each group, and treatment with each concentration was repeated three times, with the water treatment as the control group. The breeding boxes were placed in a climate-controlled chamber under laboratory conditions at 26 ± 1 °C, with 65 ± 10% relative humidity (RH). The results were examined at 24 h and 48 h. Larvae were considered dead if they did not move when touched with a brush. Stimultaneously, the toxicity of THI (with 0.1% Tween 80) and THI@M to MA was measured for comparison.

### Contact toxicity study

THI@BSA@FITC·NPs were dispersed in water and diluted to 50 mg/mL, and the larvae were treated by the abovementioned drug membrane contact method and feed mixed drug method. The distribution of THI@BSA@FITC·NPs on the surface of the larvae was observed by fluorescent stereo microscopy after treatment with the drug membrane contact method. The larvae treated with the feed mixed drug method were dissected to obtain transverse tissue sections and intestinal tissue sections, and the distribution of THI@BSA@FITC·NPs in vivo was observed by fluorescence microscopy.

To further examine how THI@BSA·NPs enter the body through the epidermal structure of larvae, the THI@BSA·NPs was dispersed in a solution of Coomassie brilliant blue dye, and the staining of the larval valve with the extension of treatment duration was observed. Coomassie brilliant blue G-250 (100 mg) was dissolved in 95% ethanol (50 mL) and 85% phosphoric acid (100 mL), and deionized water was added to obtain a final volume of 200 mL. Then, 1 mL of the dye solution was diluted to 5 mL with deionized water and filtered if precipitation occurred. THI@BSA·NPs (250 mg) was dispersed in 5 mL of PBS, and 5 mL of diluted dye solution was added. After 20 min, the filter paper was wetted with the above dye solution and placed in a petri dish. Then, three larvae were placed on the treated filter paper and allowed to crawl. Then, the larvae were removed at 30 min, 1 h, and 2 h. The staining of the larval valve was observed by a super depth-of-field 3D microscope.

### Stomach toxicity study

The transport efficiency of THI@BSA·NPs inside the larvae was tested according to a previously reported method with modifications [41]. The THI@BSA·NPs diluent solution (0.2 mL) at a concentration of 500 μg/mL was added into artificial feed (2 g), and the feed was put into a breeding box. Larvae of MA were placed into the box after food abstinence for 24 h, and ten larvae were treated in each group. The larvae were removed at 12 h and 24 h, frozen at − 20 °C for 6 h, and cut at specified positions after freezing. The foregut of the larvae is located in the head (the first and second valves of the larvae), the hindgut is located in the tail (the last two valves of the larvae); the rest of the body is the midgut. The larvae were cut into three parts according to the above positions, and then, the larval tissues were collected, stored by category, and treated with protease solution (in a water bath at 37 °C for 20 min). The larval tissues (approximately 2.5 g) were placed into centrifuge tubes, supplemented with 10 mL of purified water and soaked for 20 min. Then, 10 mL of acetonitrile was added, and ultrasonic oscillation was performed for 20 min. Then, 3 g of NaCl was added, and oscillation was performed for 1 min. The mixture was then centrifuged at 4000 rpm for 5 min. Subsequently, 1 mL of supernatant was transferred into a purification column (30 mg of PSA; 150 mg of MgSO4), oscillated for 2 min, and centrifuged at 4000 rpm for 5 min. The THI content was determined by measuring the supernatant with a UV spectrophotometer. For comparison, the transport efficiency of THI@M inside the larvae was also measured by this method.

### Transport efficiency in pine tree trunks

Pine trees (*P. densiflora* Sieb. et Zucc.) with a diameter at breast height of 20 cm were selected as the experimental objects in the Taishan forest area, Daiyue District, Tai'an city, Shandong Province, China (117.05443 east longitude, 36.215304 north latitude, 244.7 m above sea level). A hole with a diameter of 5 mm and a depth of 4 cm at was drilled by an electric drill the base of the tree trunk at 10 cm from the ground at a 45-degrees downward angle. The solutions of THI@BSA·NPs and THI@M (200 μg/mL, 10 mL; 100 μg/mL, 20 mL; 50 μg/mL, 40 mL) were injected into the hole. After 3 days, sampling was conducted at 0–50, 50–100, 100–150, and 150–200 cm above the injection hole. Due to the biological characteristics of MA, most of the larvae live in the trunk between 1.5 and 2 m above the ground. At the time of sampling, the outer bark was removed, and 10 g of tissue was obtained from a depth of 1–3 cm. After the test was completed, the holes in the pine trees were blocked with the moist soil to protect the pine tree.

Three 25-cm-long pine trunks (*P. densiflora* Sieb. et Zucc.) were picked, and the trunks were placed upright. Three diluent solutions were added dropwise into the center of the trunk. After 3 h, each 25-cm trunk was cut evenly into five parts, and each part was further cut into wood strips and put into a grinder to obtain a wood powder.

The wood powder (approximately 6 g) was placed into a centrifuge tube, and protease solution (bath at 37 °C for 20 min) was added. The pretreatment method was conducted as described above. The THI content was obtained by measuring the supernatant with a UV spectrophotometer.

### Statistical analysis

All experiments in this work were repeated three times, and statistical analysis of the data was performed by analysis of varoamce (ANOVA) using SPSS 20. All graphical data are reported as the mean ± standard deviation (SD). Significance levels were set at * *p* < 0.05.

## Results and discussion

BSA is non-toxic as a biological material with high biocompatibility, and is low-cost and with high drug loading, BSA is suitable for encapsulating various small molecule compounds, is an environmentally friendly carrier. Based on these advantages, BSA was designed as a carrier for pesticides and injecting into trees to control trunk-boring pests in this study. Even if BSA is a biological material, safety tests are still very necessary. Before the study, the safety assessment of BSA to trees was conducted. Different concentrations of BSA solutions were injected into the pine trees, and the health of the pine trees was investigated after 60 days. The pine trees under each concentration grew normally, and the tree vigor and leaves were not affected by BSA treatment. It showed that the development of BSA as a pesticide carrier had good safety for trees.

### Preparation of THI@BSA·NPs and loading efficiency

**Fig. 2:**
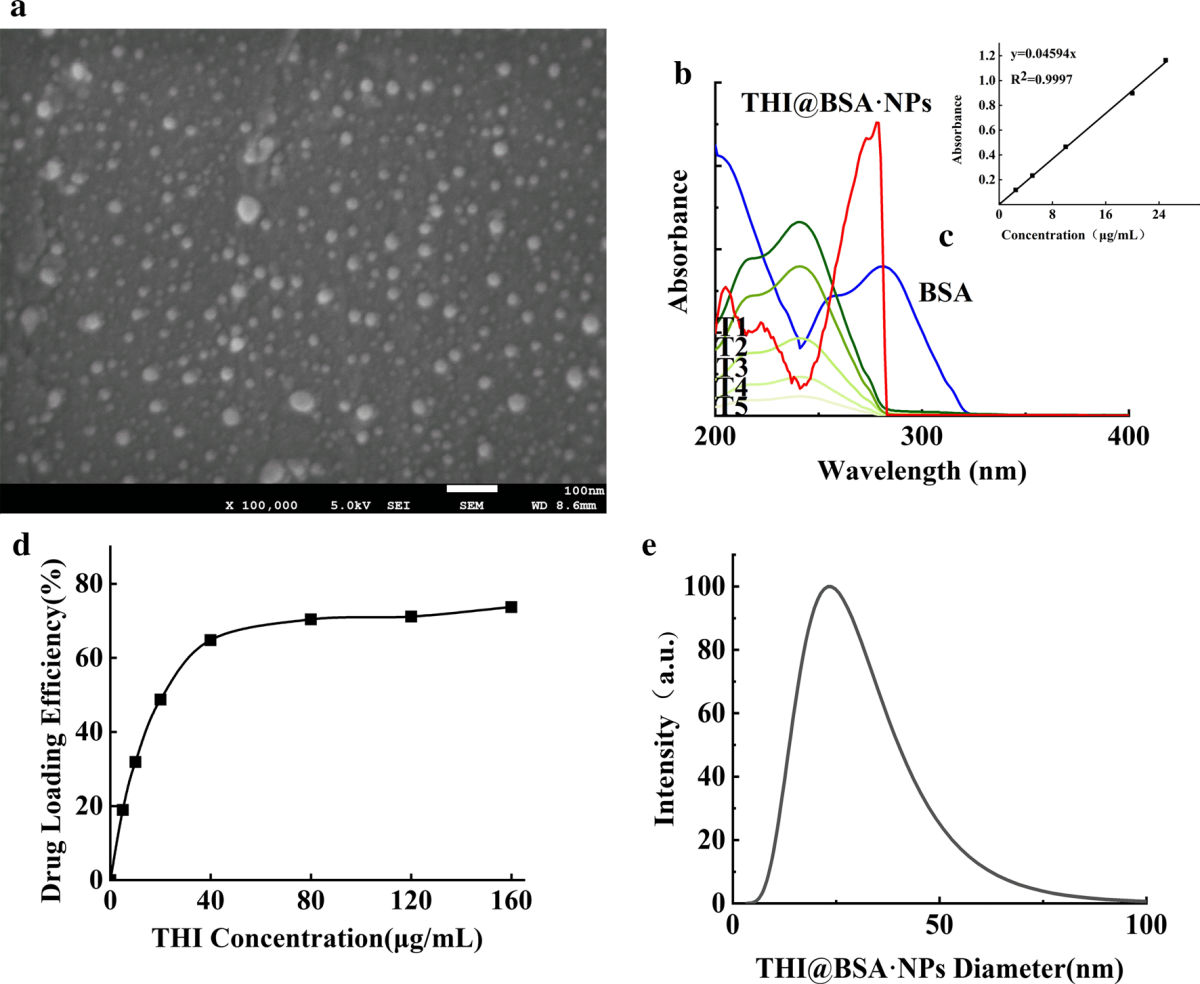
**a** SEM images of THI@BSA·NPs, **b** the UV–vis spectra of THI@BSA·NPs, BSA, and different concentration of THI, **c** the standard curve of THI, **d** particle size distribution of THI@BSA NPs, **e** the drug loading efficiency of THI@BSA·NPs with different concentration of THI

To study the structure of the synthesized THI@BSA·NPs, SEM images were used to characterize the final products. As shown in Fig. 2a, the nanoparticles were uniformly spherical and evenly dispersed in the field of view. The surface of the nanoparticles was smooth, and no holes were formed. As shown in Fig. 2b, the average particle size was approximately 23 nm, and the particle size distribution showed a single peak, indicating that the particle size distribution of THI@BSA·NPs was concentrated and that no other by-product had been produced. Continuous sampling within 30 days showed that the particle size and zeta potential of the nanoparticle powder did not fluctuate greatly, indicating that the prepared nanoparticles had good stability (Additional file 1: Figure S1).

The calibration curve of THI was linear in the concentration range of 2–25 μg/mL, the regression equation was y = 0.04594x, and the correlation coefficient was 0.9997 (Fig. 2c). The loading efficiency of THI@BSA·NPs was calculated by measuring the content of unencapsulated THI after the solvent was completely evaporated. As shown in Fig. 2d, with increasing concentrations of THI acetonitrile solution, the loading efficiency gradually increased. When the concentration exceeded 80 μg/mL, the loading efficiency was basically stable and stopped increasing. When the THI concentration reached 40 μg/mL, the optimal loading efficiency was approximately 64.8%. The above results showed that the THI@BSA·NPs prepared by encapsulating THI with BSA had a uniform nanostructure and an ideal THI loading efficiency.

### Release performance

**Fig. 3:**
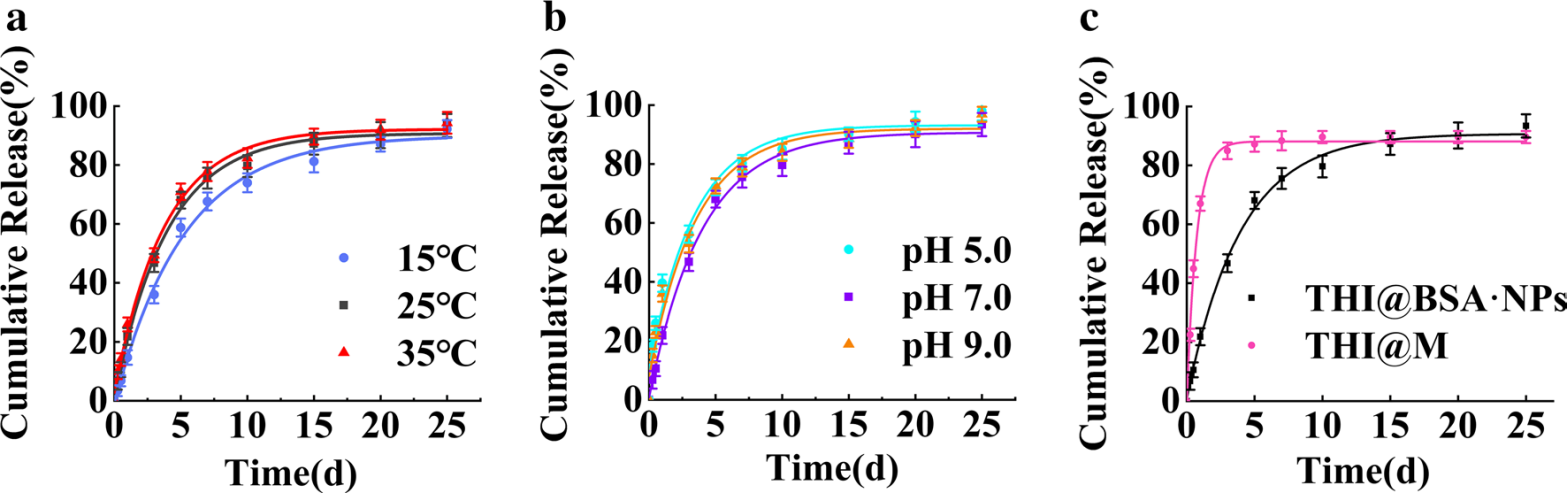
THI@BSA·NPs release curves **a** with different temperature, **b** pH, and **c** the THI@M release curves

To study the controlled-release properties of THI@BSA·NPs, the release behaviors of THI@BSA·NPs were investigated in PBS at different temperatures and pH conditions (Fig. 3), and compared with those of the commercial THI microcapsule suspension (THI@M). The temperature and pH selected in this study represented the temperature and acid–base range under natural conditions, and the physiological conditions of MA. At a pH of 7.0, the cumulative release durations were over 15 d at 15 °C, 25 °C, and 35 °C. The trends of the three release curves were basically the same, and the entire release process was stable. No sudden release occurred, and the cumulative release amounts under the three temperature conditions were approximately 93%. As the temperature increased, the release rate of THI@BSA·NPs increased to a certain extent, but the cumulative release amounts under the three temperature conditions were similar. The above results indicated that temperature conditions could affect only the release rate of THI@BSA·NPs, and did not affect the cumulative release of THI from THI@BSA·NPs. Next, at 25 °C., the release behaviors of THI@BSA·NPs were investigated in PBS at pH 5.0, 7.0 and 9.0. Under these three pH conditions, the cumulative release durations of THI@BSA·NPs were over 10 days. The pH 5.0 treatment group had the highest cumulative release (97.9%), followed by the pH 9.0 treatment group (96.8%), and the lowest cumulative release (93.4%) was observed for the pH 7.0 treatment group. Under the three pH conditions, the trends of the three release curves were also the same, and the release process was relatively stable; no sudden release occurred. These results showed that the appropriate acid–base conditions would promote the release of THI and increase the cumulative release amount. This may be related to the destruction of the structure of the protein under acid–base conditions, as more THI was released from the incomplete shell structure.

Finally, the release behavior of THI@M was investigated under the same conditions (25 °C, pH 7.0). After 3 days of treatment, THI@M reached a state of release equilibrium, with a cumulative release of approximately 90.0%. The above results show that THI@BSA·NPs had a better slow-release performance, a longer release duration, and a higher cumulative release amount, which is conducive for long-term control of pests. The development of THI@BSA·NPs might effectively reduce the frequency of application while ensuring a control effect.

The light-sensitive pesticide avermectin has a half-period of about 12 h in the soil, and even less than 6 h on the leaves. Li reported that the lignin was used as delivery system encapsulated avamectin to prepared nano-pesticide. The nano-pesticide had good slow-release performance and protected the active ingredients from photolysis. The results showed that under ultraviolet light, the half-period of avamectin was prolonged by 7.35 times compared with the original avamectin [26]. In this study, the THI@BSA·NPs had good sustained-release properties and could continuously release the active ingredients for more than 10 days. So, we speculate that the half-period of THI@BSA·NPs longer than traditional formulation. However, the half-period of THI@BSA·NPs still needs to be further explored.

### Toxicity bioassay

**Fig. 4:**
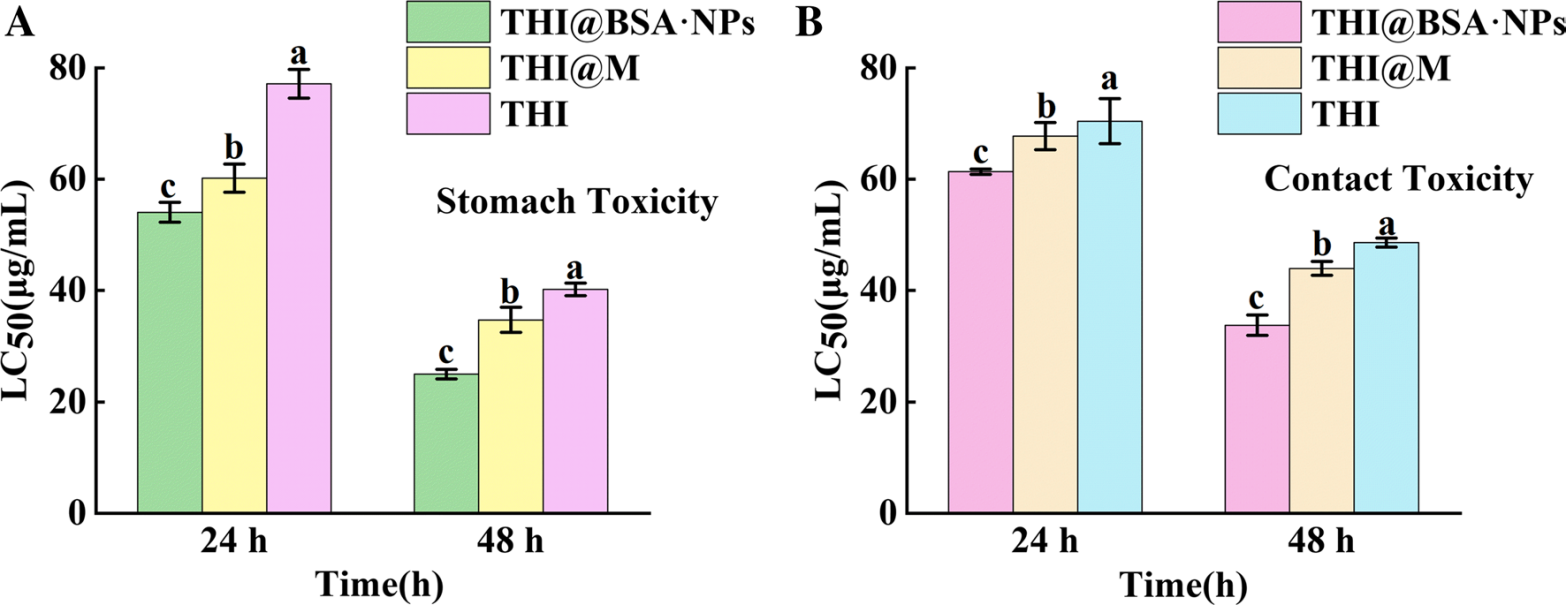
The stomach toxicity and contact toxicity of THI@BSA·NPs, THI and THI@M, bars with the same letters show no significant differences (LSD test, *p* < 0.05)

By determining the contact toxicity and stomach toxicity of THI@BSA·NPs toward the 3rd instar larvae of MA, whether the BSA nanocarriers had an effect on the bioactivity of THI was evaluated. As shown in Fig. 4, for the contact toxicity of THI@BSA·NPs against larvae at 24 and 48 h, the LC_50_ was 61.8 and 35.6 μg/mL, respectively; for the stomach toxicity at 24 and 48 h, the LC_50_ was 55.1 and 25.9 μg/mL, respectively. The toxicity of THI was also determined for comparison. for the stomach toxicity against larvae at 24 h and 48 h, the LC_50_ was 74.5 and 39.0 μg/mL, respectively the statistical data analysis was listed at Additional file 1: Tables S1 and S2. The results suggested that the toxicity of THI@BSA·NPs at 24 and 48 h was significantly higher than that of THI in the two insecticidal modes. In the stomach toxicity experiment, the larvae were acclimated to drilling into artificial feed, and some active ingredients came in contact with the larval surface. This behavior explained that the stomach toxicity in this study included a certain contact toxicity, which was one of the reasons why the stomach toxicity was much higher than the contact toxicity. These results were consistent with previous research results. Kah summarized previous research results and found that in 21 related reports, the toxicity of nanopesticides was 2.0 times higher than that of pure active ingredients [42], and nanocarriers could improve the insecticidal activity of active ingredients on multiple levels. Next, the performance of THI@BSA·NPs in the two insecticidal mechanisms was studied separately.

### Contact toxicity

**Fig. 5:**
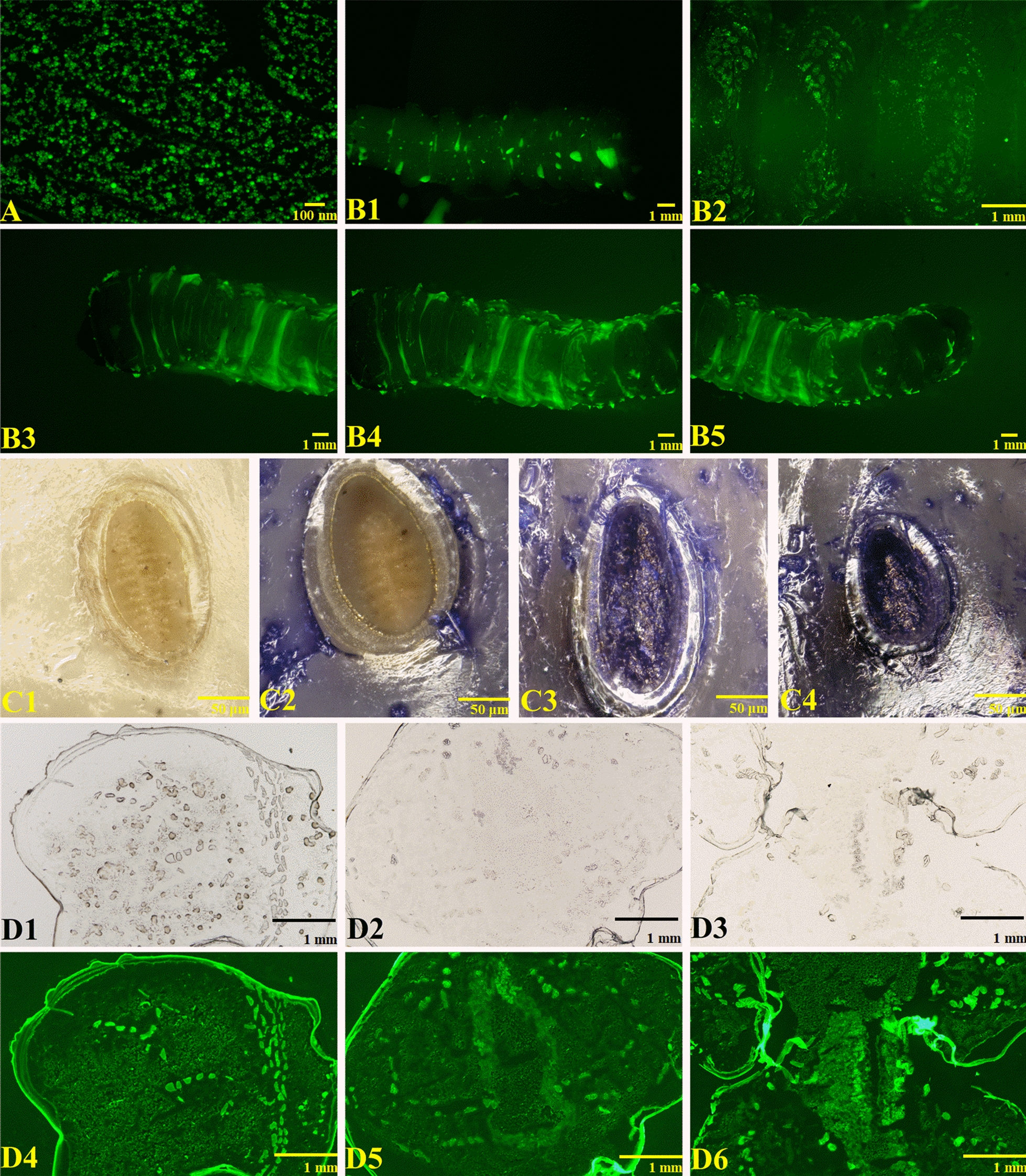
**a** The fluorescent image of THI@BSA@FITC·NPs, the distribution of THI@BSA@FITC·NPs on the larval body side (**b1**), the abdomen (**b2**), and the head (**b3**), body (**b4**), tail (**b5**), (**c1**–**c4)** the valve changed with treatment time, (**d1**–**d3**) the microscopic images of larval cross section, (**d4**–**d6**) the fluorescent distribution of THI@BSA@FITC·NPs on larval cross section

There are three main steps by which contact pesticides exert their insecticidal effects: first, they come in contact with and remain on the surface; then, they penetrate the epidermis structure; and finally, they reach the target site. Due to the nanoscale particle size and large specific surface area of nanopesticide formulations, they could improve the coverage of, adhesion to and permeation into pests compared with conventional formulations [43]. Therefore, the distribution of the fluorescence of THI@BSA@FITC·NPs on the surface of the larvae and its coverage were examined to understand the performance of THI@BSA·NPs on the surface of the larvae (Fig. 5). As shown in Fig. 5b, there was some fluorescence distributed in the head, body and tail. Compared with the heads and tails of the larvae, the fluorescence distribution in the bodies was more uniform, and the fluorescence intensity was stronger. This might be related to the greater activity of the larval head and tail, compared with the body; the swing range of the tail and the head is large, causing the attached THI@BSA@FITC·NPs to fall off. In addition, the fluorescence was more concentrated on the internodes and small protrusions on the surface of the larvae, indicating that the nanoformulation had a stronger ability to adhere to these uneven structures than to the smooth surface, and more THI@BSA@FITC·NPs remained by the holes on the internodes. In addition, fluorescence distribution was observed on each of the small rosary warts that made up the step blisters (ambulatory ampullae) for crawling on the larval abdomen. As the larvae crawl mainly by using the step blisters (ambulatory ampullae) of the abdomen, this part had a long contact duration with the THI@BSA@FITC·NPs, indicating that the longer the contact duration is, the stronger the adhesion of THI@BSA@FITC·NPs on the larval surface. These phenomena proved that, as a nanopesticide delivery system, THI@BSA·NPs exhibited good coverage and adhesion to pests, which could allow the active ingredients to come in contact with and stay on the larval surface.

It is important to study how nanoparticles penetrate the epidermal structure of pests to better understand the mechanism underlying the increase in contact toxicity. Coomassie brilliant blue-stained THI@BSA·NPs were used to explore the main mechanism by which the nanoparticles penetrated the epidermal structure into the larvae. One phenomenon caught our attention (Fig. 5c) during analysis of the staining results. The longer the larvae were exposed to the stained THI@BSA·NPs, the more intensely the valve was stained on both sides of the larval body, indicating that an increasing number of THI@BSA·NPs remained at the valve. The valve is constantly opened or closed when the larvae breathe, and the nanoparticles could easily pass through the valve and enter the body. Perhaps the nanoparticles could penetrate the epidermal structure or interstitial membrane to directly enter the larvae to exert insecticidal activity; however, the valve channels were needed when a large number of nanoparticles entered the body.

Finally, whether THI@BSA·NPs could successfully enter the larvae still needed to be verified. THI@BSA@FITC·NPs were used to treat the larval surface, and then, the treated larvae were dissected to obtain transverse sections and observed under a fluorescence microscope (Fig. 5d). It could be seen that fluorescence was uniformly distributed on the entire cross-section. A mass of dispersed AVM@BSA@FITC·NPs was clearly observed, indicating that the nanoparticles could enter the larvae through the epidermal structure and move further into the center of the larval body to deliver the AVM to the target site. The entire epidermal structure emitted bright fluorescence, indicating that a large number of THI@BSA@FITC·NPs were enriched in the surface layer and had not yet entered the larvae. These results were also verified in our previous toxicity bioassay. Therefore, THI@BSA·NPs exhibited higher contact toxicity than THI.

### Stomach toxicity

**Fig. 6:**
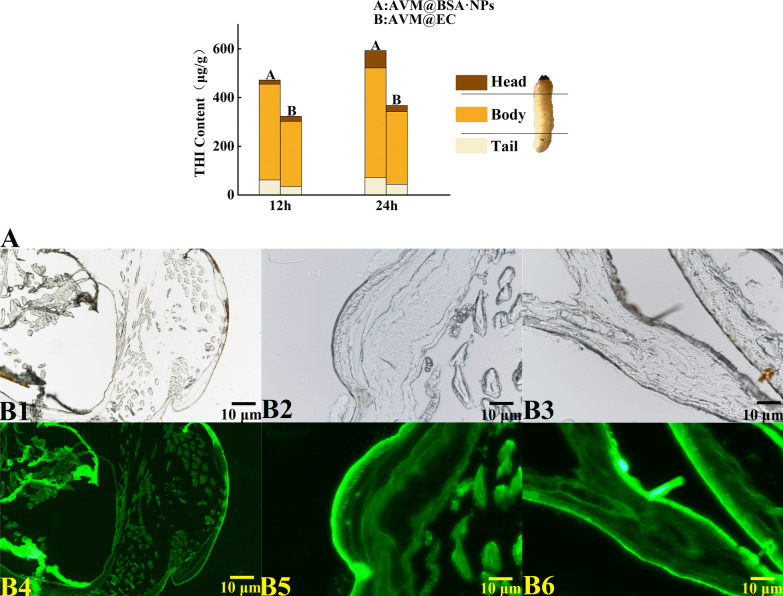
**a** Total content and proportion distribution of THI inside larvae, **b1–b3** the microscopic images of larval digestive system, **b4**–**b6** the fluorescent distribution of THI@BSA@FITC·NPs on larval digestive system

The efficiency of transport and conduction could be enhanced significantly, and the insecticidal toxicity could be accelerated, because the small particle size pf the nanoparticles improved the dispersal and permeability [44]. Therefore, the mechanism for the acceleration of stomach toxicity could be revealed (Fig. 6a) by comparing the transport efficiencies of THI@BSA·NPs and THI@M in the body. The total THI content detected in the THI@M treatment group was much smaller than that in the THI@BSA·NP treatment group. After 24 h of treatment, the THI levels in the head, body and tail of the THI@M treatment group were 0.11, 0.43, and 0.09 mg, respectively, and the total THI content was 0.63 mg. The THI levels in the head, body and tail of the THI@BSA·NP treatment group were 0.09, 0.62 and 0.15 mg, respectively, and the total THI content was 0.86 mg. After 48 h of treatment, the THI content in each treatment group increased, and the distribution trend was basically the same. In THI@BSA·NPs treatment, the content of THI in body and tail were 46.5% and 66.7% higher than that in THI@M, respectively, and in head was 22.2% lower than that in THI@M. The content of active ingredients detected in body and tail were higher in THI@BSA·NPs treatment, but detected in head was lower, indicating that more active ingredients were transported to farther locations. In the same treatment duration, in the THI@BSA·NP treatment group, more active ingredients were distributed in the body and tail; with prolongation of treatment duration, more THI was detected in the body and tail. This result suggests that THI@BSA·NPs could significantly increase the transport efficiency of active ingredients due to the size of the nanoparticles and the hydrophilicity of BSA itself. The midgut and hindgut, the major digestive and absorption systems of larvae, are concentrated in the middle and rear parts of the larvae. The higher the transport efficiency is, the greater the amount of active ingredients that could be absorbed, further improving the bioactivity of THI.

The larvae were fed artificial feed mixed with THI@BSA@FITC·NPs, and then, the treated larvae were collected and dissected to obtain intestinal tissue sections. Then, the fluorescence distribution of intestinal tissues was observed by fluorescence microscopy (Fig. 6b). Fluorescence distribution was also observed in the intestinal tissues, indicating that under oral feeding, THI@BSA@FITC·NPs had high transport efficiency inside larvae and could be smoothly transported to intestinal tissues. The villi inside the intestinal wall could clearly be seen, and the fluorescence intensity inside the intestinal wall was the strongest due to the adhesion of THI@BSA@FITC·NPs to these villi. Fluorescence was also observed on the fat particles outside the intestine, indicating that the intestine effectively absorbed THI@BSA@FITC·NPs and quickly converted them into nutrients. Shen's research showed that nanocarriers could efficiently penetrate insect midgut cells to reach the target site, thereby improving activity [28]. This phenomenon revealed that a small number of THI@BSA@FITC·NPs could escape directly through the intestinal tissue cells due to the nanoscale size of the particles.

### Transport efficiency tree trunks

**Fig. 7:**
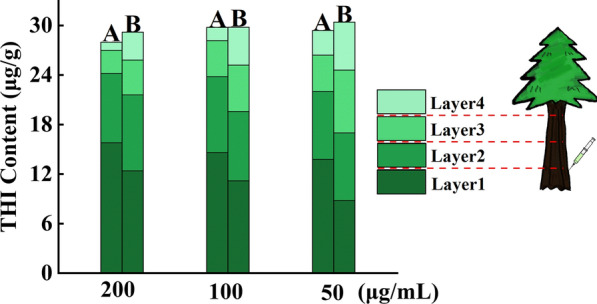
Total content and proportion distribution of THI in the trunk of alive pine tree

Since the larvae live in the trunk of the pine tree, which acts as a natural barrier against chemical control, THI@BSA·NPs were designed to be injected into the trunk during application; thus, the transport efficiency in the trunk of the pine tree would directly affect the application. Therefore, at the end of this study, the transport efficiencies of THI@M and THI@BSA·NPs in the trunk were compared (Fig. 7). As shown in the figure, the transport efficiency of THI@BSA·NPs was significantly higher than that of THI@M at the same concentration. The distribution of THI in the THI@BSA·NP treatment group was more even than that of THI@M; when the concentration was 50 mg/mL, the proportions of the four layers were 28.94%, 26.97%, 25.00% and 19.09% in the THI@BSA·NP treatment group and 45.41%, 26.97%, 14.50%, and 9.84% in the THI@M treatment group. On the other hand, as the concentration decreased, the transport efficiencies of the THI@BSA·NP treatment group and the THI@M treatment group improved. In the THI@M treatment group, the THI levels in layer 4 were 1.0, 1.6 and 3.0 mg/g when the concentrations were 200, 100, and 50 mg/mL, respectively, and in the THI@BSA·NP treatment group, the levels were 3.4, 4.6, and 5.8 mg/g, respectively. This showed that with decreasing concentration, greater amounts of active ingredients could be transported farther away, and the migration distance could be effectively increased. THI@BSA·NPs are nanosized particles with high dispersibility and a long migration distance in water, and all these features would allow the active ingredients reach the site where the target organism resides more efficiently, improving the application efficiency and reducing the pesticide application amount. In applications, the migration distance in the trunk could be increased by appropriately diluting the particles without affecting the control effect of THI to achieve the maximum performance.

**Fig. 8:**
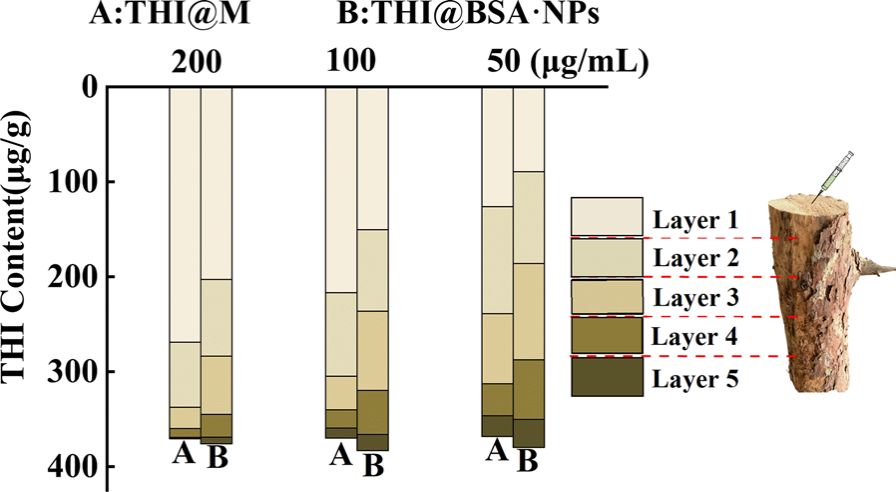
Total content and proportion distribution of THI in the trunk of dead pine tree

THI@BSA·NPs were considered for treatment of diseased wood to reduce the cost of transporting the diseased wood in high-altitude areas to resettlement sites. The transport efficiencies of different concentrations and different formulations were also studied, and the distribution of nanoparticles in the trunks of dead trees was explored. The results, as shown in Fig. 8, were the same as those for live trees. When the concentration was 200 μg/mL, the THI content in the layers was 89.4, 96.5, 101.7, 62.6 and 29.7 μg/g, and the corresponding values in the THI@M group were 126.0, 112.9, 73.9, 33.6, 21.9 μg/g. In THI@BSA·NPs, the proportions of layer 1 to layer 5 were 23.5%, 25.4%, 26.8%, 16.5% and 7.8% respectively under 50 μg/L, and the proportions were 34.2%, 30.6%, 20.1%, 9.1% and 5.9% respectively, the distribution of THI@BSA·NPs in the trunks of dead trees was more even. Obviously, driven by the same concentration and the same amount of water, the migration distance of THI@BSA·NPs was longer, and the THI concentration detected at farther layer was higher, indicating that THI@BSA·NPs has a higher transport efficiency. On the other hand, when the content of active ingredients were same, the lower the concentration was, the more uniform the distribution.

Generally speaking, the smaller the particle size of the nanoparticles, the larger the specific surface area and the higher the loading efficiency [45, 46]. The size of the nanoparticles we prepared was 23 nm, which was less than 100 nm. Compared with the microcapsules, the size was smaller, so the loading efficiency was higher. On the other hand, the particle size of 23 nm allowed nanoparticles to easily pass through the conducting tissue of trees, and the high transport efficiency of THI @ BSA·NPs enables the active ingredients to reach the survival sites of the trunk-boring pests more efficiently. At the same time, smaller particle size could effectively improve the coverage, adhesion and permeability on pests of pesticide [43, 47], so the nanoparticles could efficiently penetrate the epidermal structure to improve contact toxicity. The high transport efficiency inside larvae allowed the active ingredients to reach the site of action efficiently and exert insecticidal toxicity. The results of transport efficiency also verified this conclusion. Finally, the great slow-release performance enabled THI@BSA·NPs to control trunk-boring pests for a long time. Therefore, the nano-pesticide THI@BSA·NPs prepared had great control efficiency on trunk-boring pests. At present, trunk injection is still the main method to control trunk-boring pests with the characteristics of highly targeted and little impact on non-target organisms. However, the control effect is limited by the low transportation efficiency and shorter release period inside trunk. In this study, biological material BSA were used as pesticide carrier for the preparation of THI@BSA·NPs, which has the characteristics of high efficiency and sustained release. The good transport efficiency inside trees ensured that the active ingredients reached the survival site of the target organism. Meanwhile, nanoparticles could enhance the coverage, adhesion, and penetration of active ingredients [48], so, compared with traditional formulation, THI@BSA·NPs could effectively improve the stomach toxicity and contact toxicity of THI. Based on the above conclusions, we believe that THI@BSA·NPs has better control efficiency for trunk-boring pests than traditional formulations. Currently, the control of trunk-boring pests is still a difficult problem. The nano-pesticide, THI@BSA·NPs prepared by us, has low cost, easy synthesis, high biocompatibility, and low environmental impact. At the same time, compared with traditional formulations, great slow-release performance and higher control efficiency made THI@BSA·NPs have broad application prospects in the control of trunk-boring pests.

## Conclusion

In this study, a nanoparticle with high bioactivity against TBPs was successfully synthesized and verified as having an excellent control effect. THI@BSA·NPs, which are particles with a size of approximately 23 nm and a loading efficiency of up to 70.4%, were stable under different temperatures and pH values and could release THI stably over 15 d, and the cumulative release exceeded 90%. By using various dyeing techniques, nanoparticle distributions and sections were observed. The nanoparticles showed a better distribution range on, adhesion to, and penetration of the larval surface, and at the same time, they exhibited a higher transport efficiency inside larvae, ensuring that the active ingredients were ingested. The contact and stomach toxicities of THI@BSA·NPs increased by 25.9% and 33.7%, respectively, compared with those of THI. Finally, compared to the commonly used formulation in production, THI@BSA·NPs had a higher transport efficiency in live or dead trees and could ensure that the active ingredients reached the target parts. In summary, the THI@BSA·NPs synthesized in this study could effectively control TBPs.

## Supplementary information

**Additional file 1: Table S1.** The stomach toxicity of THI@BSA·NPs, THI and THI@M. **Table S2.** The contact toxicity of THI@BSA·NPs, THI and THI@M. **Fig. S1.** The particle size and Zeta potential of THI@BSA·NPs under different temperature conditions within 30 days.

## Data Availability

All data generated or analyzed during this are included in this published article.
