# Supplementary material for: Self-assembled bovine serum albumin nanoparticles as pesticide delivery vectors for controlling trunk-boring pests
Source: J Nanobiotechnology. 2020 Nov 10;18:165. doi: 10.1186/s12951-020-00725-z (PMC7653776; doi:10.1186/s12951-020-00725-z)
Supplement: Supplementary file 1 — Additional file 1: Table S1. The stomach toxicity of THI@BSA·NPs, THI and THI@M. Table S2. The contact toxicity of THI@BSA·NPs, THI and THI@M. Fig. S1. The particle size and Zeta potential of THI@BSA·NPs under different temperature conditions within 30 days. [file 12951_2020_725_MOESM1_ESM.docx]

**Additional file**

**Self-Assembled Bovine Serum Albumin Nanoparticles as Pesticide Delivery Vector for Controlling Trunk-boring Pest**

Chenyu Su^a^, Shanshan Liu^a^, Shenghan Cao^a^, Shuyan Yin^a,b^, Chenggang Zhou^a,b^, Shangkun Gao^a,b^, Chunyan Jia^d^, Yingchao Ji^a,b*^, Yanxue Liu^c *^

*^a^ College of Plant Protection, Shandong Agricultural University, Tai’an, Shandong 271018, P. R. China;*

*^b^ Shandong Research Center for Forestry Harmful Biological Control Engineering and Technology, Shandong Agricultural University, Tai’an, Shandong* *271018, P. R. China;*

^c^ College of Animal and Veterinary Medicine, Shandong Agricultural University, Tai’an, Shandong 271018, P. R. China;

^d^ Taishan Scenery and Scenic Spot Area Management Committee, Tai'an, Shandong 271000, P. R. China.

*Corresponding Author’s Email: jiyc2018@sdau.edu.cn, [liuyouyou2018@163.com](mailto:liuyouyou2018@163.com)

Number Pages: 4

Number Tables: 2

Number Table: 1

**Table of Content**

Table S1 The stomach toxicity of THI@BSA·NPs, THI and THI@M S3

Table S2 The contact toxicity of THI@BSA·NPs, THI and THI@M S4

Fig. S1 The particle size and Zeta potential of THI@BSA·NPs S4

Table S1 The stomach toxicity of THI@BSA·NPs, THI and THI@M

| Treatment | Repeat | Time | Toxicity regression equation | LC_50_(μg/mL)（95%CI） | Chi-square | Coefficient of Association |
| --- | --- | --- | --- | --- | --- | --- |
| THI@BSA·NPs | 1 | 24 h | y=1.9512+1.7455x | 55.8074 | 11.0955 | 0.7883 |
|  |  |  |  | 39.5673-97.9574 |  |  |
|  |  | 48 h | y=2.7539+1.6025x | 25.8773 | 5.3687 | 0.9375 |
|  |  |  |  | 18.6397-36.2499 |  |  |
|  | 2 | 24 h | y=2.9003+1.2221x | 52.2503 | 5.8903 | 0.7115 |
|  |  |  |  | 34.0759-112.4379 |  |  |
|  |  | 48 h | y=3.3613+1.1849x | 24.1564 | 4.8017 | 0.9032 |
|  |  |  |  | 15.2506-37.6709 |  |  |
| THI | 1 | 24 h | y=2.9539+1.0928x | 74.5370 | 4.8265 | 0.6803 |
|  |  |  |  | 43.7357-249.9233 |  |  |
|  |  | 48 h | y=2.9345+1.2980x | 39.0180 | 2.1428 | 0.9575 |
|  |  |  |  | 26.6823-67.8825 |  |  |
|  | 2 | 24 h | y=2.9724+1.0664x | 79.6983 | 1.8725 | 0.9417 |
|  |  |  |  | 45.6135-299.9038 |  |  |
|  |  | 48 h | y=3.1909+1.1196x | 41.2875 | 1.7065 | 0.9577 |
|  |  |  |  | 26.7330-83.0804 |  |  |
| THI@M | 1 | 24 h | y=2.7824+1.2339x | 62.6877 | 4.1051 | 0.7232 |
|  |  |  |  | 39.7527-152.4770 |  |  |
|  |  | 48 h | y=2.9082+1.3832x | 32.5341 | 1.9436 | 0.9651 |
|  |  |  |  | 22.7427-50.9013 |  |  |
|  | 2 | 24 h | y=3.1754+1.0363x | 57.6387 | 2.8312 | 0.9265 |
|  |  |  |  | 34.9415-164.2289 |  |  |
|  |  | 48 h | y=3.2544+1.1136x | 36.9395 | 2.7867 | 0.9344 |
|  |  |  |  | 23.9198-70.0847 |  |  |

Table S2 The contact toxicity of THI@BSA·NPs, THI and THI@M

| Treatment | Repeat | Time | Toxicity regression equation | LC_50_(μg/mL)（95%CI） | Chi-square | Coefficient of Association |
| --- | --- | --- | --- | --- | --- | --- |
| THI@BSA·NPs | 1 | 24 h | y=2.3370+1.4869x | 61.7967 | 3.4058 | 0.7795 |
|  |  |  |  | 41.4223-126.3269 |  |  |
|  |  | 48 h | y=2.7491+1.4506x | 35.6261 | 3.5498 | 0.9536 |
|  |  |  |  | 25.2847-56.1376 |  |  |
|  | 2 | 24 h | y=2.5857+1.3531x | 60.8528 | 2.6768 | 0.9336 |
|  |  |  |  | 39.8911-132.4461 |  |  |
|  |  | 48 h | y=3.0805+1.2757x | 31.9613 | 3.3583 | 0.9385 |
|  |  |  |  | 21.7099-51.8486 |  |  |
| THI | 1 | 24 h | y=2.2249+1.4824x | 74.4672 | 3.5663 | 0.7983 |
|  |  |  |  | 47.8698-174.8319 |  |  |
|  |  | 48 h | y=3.1767+1.0764x | 49.4069 | 2.2142 | 0.9404 |
|  |  |  |  | 31.0543-117.2973 |  |  |
|  | 2 | 24 h | y=2.6807+1.2730x | 66.3674 | 5.4358 | 0.7356 |
|  |  |  |  | 42.0008-163.3181 |  |  |
|  |  | 48 h | y=3.0605+1.1549x | 47.7860 | 3.2215 | 0.9327 |
|  |  |  |  | 30.9287-102.4055 |  |  |
| THI@M | 1 | 24 h | y=2.8287+1.1761x | 70.1857 | 2.8514 | 0.9363 |
|  |  |  |  | 42.8063-198.9193 |  |  |
|  |  | 48 h | y=2.8579+1.2944x | 45.1761 | 2.7323 | 0.9496 |
|  |  |  |  | 30.5421-84.7459 |  |  |
|  | 2 | 24 h | y=2.9977+1.1033x | 65.2810 | 3.6325 | 0.9113 |
|  |  |  |  | 39.5679-189.4747 |  |  |
|  |  | 48 h | y=3.6183+0.8475x | 42.6993 | 2.1148 | 0.9153 |
|  |  |  |  | 24.3485-129.9856 |  |  |



Fig. S1 The particle size and Zeta potential of THI@BSA·NPs under different temperature conditions within 30 days.
